# Cancer Biology Analysis—Tackled from Different Points of View

**DOI:** 10.3390/medicina57090937

**Published:** 2021-09-05

**Authors:** Alberto Ouro

**Affiliations:** Clinical Neurosciences Research Laboratories, Health Research Institute of Santiago de Compostela (IDIS), 15706 Santiago de Compostela, Spain; alberto.ouro.villasante@sergas.es; Tel.: +34-664326589

In the last few decades, great advances have been made in the detection and treatment of cancer, thus increasing the survival rate. The design of new biomarkers and specific drugs has been, and is of, vital importance. However, many types of cancer manage to adapt and evade anti-cancer treatments through their great capacity for acceptance and their interaction with other cells. That is why interdisciplinary cancer cell and tumor research is of vital importance. In this Special Issue, we have tried to address these facts from different areas.

Sphingolipids are complex molecules indispensable for cell architecture and are also involved in cellular signaling. Different species of sphingolipids have been characterized, varying in their structure and chain length, and in the possession of a phosphate group or not. These variations can lead to the activation of antagonistic signaling pathways and thereby determine one cell fate or another. Sphingolipids have been described as regulators of different cellular functions, many of them involved in the growth and spread of cancer cells. They have been implicated in proliferation, apoptosis, cell migration, cell invasion, inflammation and metabolism, among others. Thus far, more than 30 enzymes involved in sphingolipid metabolism have been described. Some specific inhibitors of certain enzymes have shown their efficiency against cancer cells. That is why the study of your metabolism is crucial for the development of new therapies. Gomez-Larrauri et al. have summarized the most interesting findings on sphingolipid metabolism in this section [1].

The relationship of the microbiota with the regulation and adaptation of the organism and, more specifically, the immune system has been a subject of debate in recent years. Different experiments have demonstrated the obvious relationship between the microbiota and carcinogenesis. The development of germ-free animals has been shown to be a very useful tool for studying the involvement of the microbiota in cancer. In studies on the development of spontaneous tumors in rats and mice, a higher prevalence has been observed in control animals compared to germ-free animals. The immune system is responsible for regulating the specificity and quantity of bacteria in the body. For example, the stimulation of the immune system by bacteria has been postulated as one of the possible roles of the relationship of the microbiota with the development of cancer. Mishra et al. have elegantly reviewed the most relevant studies in this field, both in spontaneous and induced tumor models [2].

Cancer cells are characterized by high and uncontrolled proliferation rates. This increased cell division leads to an increased likelihood of genetic mutations. Microsatellites are sequences of six or more nucleotides repeated in certain DNA motifs. Their length is altered during DNA replication, since they are located in loci vulnerable to errors in replication, a phenomenon known as microsatellite instability (MSI). MSIs have been described in 20% of gastric cancers, and have been stipulated as good biomarkers for prognosis and for the selection of a specific treatment. However, several studies have questioned its usefulness. Park et al. have conducted a next-generation sequencing (NGS) study to determine whether or not MSIs are a rare phenomenon in gastric cancer [3]. The authors confirmed a rare phenomenon of microsatellite instability in GISTs, irrespective of diverse genomic alterations. Moreover, Park et al. observed a relationship between mutations in exon 11 or 9 of the KIT gene with the response to treatment with imatinib. More studies are required for the establishment of a mutational pattern that allows the use of MSIs as biomarkers and a personalized selection of therapies.

Primary cutaneous lymphoma (PCL) is a subtype of non-Hodgkin lymphoma and presents a progressive clonal proliferation of B-cells, T-cells or NK-cells in the skin. Meanwhile, Mycosis fungoides (MF) is the most common lymphoproliferative disorder neoplastic T-cell with a special affinity for the skin. Both diseases share similar etiologies, making it difficult to diagnose between the two, delaying it by an average of 6 years. Gug et al. have carried out an exhaustive study of genetic clonality, providing a great variety of data that should be studied in depth [4]. Unfortunately, they have observed great similarity between both diseases when studying the number of clones of different genes between both diseases. Interestingly, they have observed differences in the receptor T-Cell gene with a high incidence in MF, which should be studied in depth.

The presence of macrophages in the microenvironment has been described as essential for the growth and spread of cancer. Macrophage polarization between the M1 (pro-inflammatory) to M2 (anti-inflammatory) subpopulations is the crucial step for their progression. However, the specific mechanisms that lead to this change are unknown. Kuo et al. have shown that the microenvironment produces oxidative stress in macrophages due to an accumulation of reactive oxygen species (ROS). The incubation of macrophages with conditioned medium demonstrates the change in polarization when studying membrane markers such as CD86 and CD206. Previous work demonstrated that endoplasmic reticulum (ER) stress participated in macrophage polarization. Kuo et al. also observed a decrease in the proteins involved in ER stress such as CHOP, ATF6 and ATF4. This study provides information of interest for the development of anticancer therapies [5].

Glioblastoma is considered a malignant tumor with a poor prognosis for the patient. Today, the only treatment that has shown any effectiveness is the surgical excision of the tumor, followed by radiation therapy and chemotherapy. However, its efficiency is low due to the aggressiveness of this type of tumor. Luteolin is abundant in peanut shells and is also found in herbs and other plants, such as thyme, green pepper and celery. Luteolin is known to be effective against obesity and metabolic syndrome and its anti-inflammatory and anti-cancer properties have been investigated. Apoptosis and autophagy are cellular mechanisms of response to abnormalities. In a piece of work by Lee and co-workers, the ability of luteolin to stimulate apoptosis and autophagy in glioblastoma cell lines has been evaluated. The authors have described that luteolin stimulates the production of active Caspase-3, leading to apoptosis. Furthermore, the authors have shown that lutein increases the expression of the proteins involved with the formation of the autophagosome, such as ATG5, with the consequent increase in complex proteins such as LC3B. This article demonstrates, for the first time, how lutein could be used in the treatment of glioblastoma [6].

In summary, the findings reported in the works published as part of this Special Issue provide relevant data in the different areas of cancer study. In addition, the data and information provided should be taken into account for the establishment of new study objectives. This will lead to a better understanding of the processes involved in carcinogenesis and the development of new therapies.

## Data Availability

Not required.
